# Should the Start of Immunosuppressive Treatment for COVID-19 Rely upon the Degree of Inflammation or the Time from Onset?

**DOI:** 10.3390/medicina61020233

**Published:** 2025-01-27

**Authors:** José María Mora-Luján, Abelardo Montero, Francesc Formiga, Manuel Rubio-Rivas

**Affiliations:** 1 Department of Internal Medicine, Parc Sanitari Hospital del Mar, 08003 Barcelona, Spain; josemaria.mora.lujan@psmar.cat; 2 Department of Internal Medicine, Bellvitge University Hospital, 08907 Barcelona, Spain; asaez@bellvitgehospital.cat (A.M.); fformiga@bellvitgehospital.cat (F.F.)

**Keywords:** COVID-19, treatment, corticosteroids, prognosis, risk factors, mortality

## Abstract

*Background and Objectives:* A COVID-19 model with a viral first-week phase and an inflammatory second phase has been proposed. It has been suggested that immunosuppressive treatment in the first week is harmful. This study aimed to analyze the potential damage of corticosteroids (CS) administered in the first week of COVID-19. *Materials and Methods:* This study was performed on a large cohort of consecutive COVID-19 patients admitted to Bellvitge University Hospital (Barcelona, Spain) from March 2020 to April 2021. Patients diagnosed with COVID-19 who were treated with 6 mg of dexamethasone a day for 10 days, and whose initiation of administration occurred within the first 2 weeks from symptom onset were included. We divided the cohort into the following two groups: patients for whom CS were initiated within the first 7 days after symptom onset vs. patients for whom CS were initiated between days 8 and 14. The degree of analytical inflammation (based on lymphocyte count, C-reactive protein, ferritin, lactate dehydrogenase, and D-dimer) upon admission was taken into account. The primary outcome was in-hospital mortality. *Results:* A total of 581 patients met the inclusion criteria. The results included, as follows: differences in age at baseline between groups (70.8 years old vs. 62.7, *p* < 0.001); moderate-to-severe dependency (11.9% vs. 4.2%, *p* = 0.003); the lymphocyte count (840 × 10^6^/L vs. 900, *p* = 0.033); D-dimer (400 ng/mL vs. 309, *p* < 0.001); and PaO_2_/FiO_2_ (290 vs. 311, *p* < 0.001). In-hospital mortality in patients who received CS in the first week of symptom onset was higher (29% vs. 12.8%, *p* < 0.001). The following risk factors were associated with higher in-hospital mortality: age (OR = 1.06, *p* < 0.001); Charlson index (OR = 1.34, *p* = 0.001); tachypnea > 20 bpm (OR = 2.58, *p* < 0.001); ≥3 high-risk criteria of inflammation (OR = 1.94, *p* = 0.012); and CS onset in the first week (OR = 2.17, *p* = 0.004). A higher PaO_2_/FiO_2_ (OR = 0.99, *p* < 0.001) and the use of remdesivir (OR = 0.53, *p* = 0.021) were identified as protective factors. However, when stratified by analytical inflammation criteria, the onset of CS in the first week did not reach statistical significance. *Conclusions:* The early administration of CS did not demonstrate a significant detrimental effect. These results highlight the need for a nuanced approach to CS therapy in COVID-19 that carefully weighs the risks and benefits based on individual patient characteristics and the severity of the inflammation.

## 1. Introduction

COVID-19 is a disease with a viral replication phase, usually lasting about 7 days, followed by an inflammatory response phase that, in some patients, can trigger acute respiratory distress syndrome (ARDS)—the so-called “cytokine storm” [1,2]. Since the publication of the RECOVERY clinical trial [3], dexamethasone has been the treatment of choice. Despite good mortality results, the optimal time to initiate it is uncertain.

Subsequent RCTs obtained similar results to those of RECOVERY, demonstrating the beneficial role of corticosteroids (CS) [4,5,6]. Thus, international guidelines recommend CS for severe COVID-19 [7,8,9]. Other studies have compared dexamethasone with other CS, such as methylprednisolone, as well as high versus low doses, with similar results in terms of mortality and disease progression [10,11]. It is well known that the immunomodulatory effect of CS occurs through two mechanisms, genomic and non-genomic. The genomic mechanism is activated at low–intermediate doses and generates a trans-repression effect (decreased production of nuclear factor k-light-chain-enhancer of activated B cells and proinflammatory cytokines) and a trans-activation effect (increased expression of anti-inflammatory molecules). On the other hand, the non-genomic mechanism is activated at high doses of CS and triggers an activation of intracellular kinase-mediated signaling cascades with anti-inflammatory effects [12,13,14]. The absence of differences between high and low doses of CS suggests that it is the genomic mechanism that plays a determining role in the pathophysiology of COVID-19. However, these studies do not evaluate the timing of CS initiation. CS, especially dexamethasone, has also been shown to have a better cost–benefit ratio than other treatments targeting COVID-19 [15].

Although the RECOVERY study did not include viral load in its endpoints, several studies have cited it with respect to preventing the early use of corticosteroids in the known viral replication phase of the virus [16,17,18]. However, studies designed to assess viral clearance in corticosteroid-treated patients showed no difference in patients who were not treated with CS [19,20]. In contrast, the RECOVERY clinical trial and subsequent trials have shown that CS are not beneficial in patients without oxygen [3,21].

In addition, several subsequent studies and meta-analyses show the benefit of corticosteroids regardless of symptom duration [22,23,24,25,26,27,28]. Stern et al., in a subset of the meta-analysis they performed, found a beneficial effect on patients for whom CS was initiated within 7 days of symptom onset [22]. Siang Kow C. et al. also performed a meta-analysis to compare high versus low doses of dexamethasone, showing improved mortality in both groups regardless of symptom duration [23]. Ssentongo et al. also found that CS regimens of different durations reduced mortality regardless of symptom duration [27].

Thus, there are conflicting data on the timing of the initiation of CS based on the number of days of symptoms. There is a widespread opinion that they are harmful when administered on the first day; however, data from multiple studies suggest otherwise. This study aimed to analyze the potential harm of CS administered in the first week of COVID-19.

## 2. Materials and Methods

### 2.1. Study Design, Patient Selection, and Data Collection

This study was performed on a large cohort of consecutive patients (all patients admitted to the hospital for COVID-19 during this period are registered in our database) admitted to Bellvitge University Hospital (a tertiary hospital in Hospitalet de Llobregat, Barcelona, Spain) due to COVID-19. From March 2020 to April 2021, 2284 patients were admitted to our hospital due to COVID-19 and included in our registry. All included patients were diagnosed by polymerase chain reaction (PCR) or rapid antigenic tests for SARS-CoV-2 taken from nasopharyngeal, sputum, or bronchoalveolar lavage specimens. Data for each patient regarding sociodemographic data, comorbidities, laboratory data, treatments, and outcomes were verified by a review of medical records.

The Ethics Committee approved the registry and database (PR 128/20).

### 2.2. Inclusion Criteria

Patients diagnosed with COVID-19 treated with standard doses of dexamethasone according to the RECOVERY study (dexamethasone at 6 mg per day for 10 days or equivalent) [3] and whose initiation of administration occurred within the first 2 weeks from symptom onset were included. Disease onset was defined as the day on which the patient presented the first COVID-19-related symptom. The degrees of analytical inflammation previously described by our group [29] were taken into account; therefore, only those patients categorized as being at a high analytical risk were included in the present study.

Specifically, the high-risk category was defined as any patient with at least 1 of the following criteria in the admission lab test: lymphopenia < 760 × 10^6^/L; CRP > 101.5 mg/L; ferritin > 1359.9 mcg/L; LDH > 394 U/L; or D-dimer > 1580 ng/mL.

### 2.3. Exclusion Criteria

Patients who did not receive CS or who received CS at other doses or for other durations than those described in the RECOVERY study were excluded [3]. Patients with nosocomial COVID-19 and those with mild or moderate degrees of analytical inflammation were also excluded. In addition, patients with autoimmune diseases were not included in this study.

### 2.4. Treatments Prescribed and Definitions of Groups

We divided the cohort into the following 2 groups: the first group included patients who were administered CS within the first 7 days after symptom onset; and the second group included patients who were administered CS between days 8 and 14 after symptom onset.

Patients concomitantly received remdesivir (RDSV), tocilizumab (TCZ), and low molecular-weight heparins (LMWH) according to the hospital protocol and medical criteria. These medications were included in the matching and their administration is described in the tables.

### 2.5. Outcomes Definition

The primary outcome of the study was in-hospital mortality. Secondary outcomes included, as follows: the composite variable of in-hospital mortality; the need for a high-flow nasal cannula (HFNC); the need for non-invasive mechanical ventilation (NIMV); the need for invasive mechanical ventilation (IMV); and intensive care unit (ICU) admission.

### 2.6. Statistical Analyses

Multiple imputations of missing data were performed. To minimize differences between groups and improve comparability, 1:1 propensity-score matching (PSM) was performed. The PSM included, as follows: sociodemographic variables (age, sex, and race); comorbidities (smoking behavior, body mass index, and Charlson index); laboratory variables at admission (PaO_2_/FiO_2_ (PAFI), ferritin, lactate dehydrogenase (LDH), C-reactive protein (CRP), lymphocyte count, and D-dimer); and treatments during admission (RDSV, TCZ, and LMWH).

Categorical variables were expressed as absolute numbers and percentages. Continuous variables were expressed as mean plus standard deviation (SD) in the case of parametric distribution or as median [IQR] in the case of non-parametric distribution. Differences among groups were assessed using the chi-squared test for categorical variables and the T-test or Mann–Whitney test as appropriate for continuous variables. *p*-values < 0.05 indicated statistical significance.

For the study of risk factors for in-hospital mortality, binary logistic regression was performed. Those variables with *p* < 0.10 in the univariate study plus age and sex were introduced in the multivariate model. The statistical analysis was performed by IBM SPSS Statistics for Windows, Version 26.0. Armonk, NY, USA: IBM Corp.

## 3. Results

### 3.1. General Data and Symptoms Between Groups

A total of 2284 patients were included in the registry by April 2021; 581 patients met the inclusion criteria for the present study, 293 patients received CS in the first week after the onset of symptoms and 288 patients received CS in the second week after the onset of symptoms.

Table 1 shows the differences between the groups. Differences were found in the baseline age between the two groups (70.8 years vs. 62.7, *p* < 0.001). Differences were also found in the degree (moderate–severe) of dependency (11.9% vs. 4.2%, *p* = 0.003), and of several comorbidities. Among these, differences between groups for arterial hypertension (64.5% vs. 49.7%, *p* < 0.001) and dyslipidemia (50.5% vs. 39.6%, *p* = 0.008) were notable. Despite the application of the PSM, these differences between the groups were still found (Table 1).

Regarding symptoms on admission, patients who received CS in the second week presented more frequently with, as follows: coughs (65.5% vs. 77.4%, *p* = 0.006); arthromyalgias (19.1% vs. 31.9%, *p* < 0.001); ageusia (13.3% vs. 22.2%, *p* = 0.005); anosmia (10.9% vs. 19.1%, *p* = 0.006); headache (12.3% vs. 19.4%, *p* = 0.018); and diarrhea (26.3% vs. 36.5%, *p* = 0.008). These results were reproducible in the matched sample (Table S1).

### 3.2. Lab Tests Between Groups

All patients had a baseline blood test compatible with the high inflammation risk category. Significant differences between groups were found in the baseline median lymphocyte count (840 × 10^6^/L vs. 900, *p* = 0.033), and D-dimer (400 ng/mL vs. 309, *p* < 0.001). High-risk criteria were similar between the groups. Differences were also found in PAFI between the groups upon admission (290 vs. 311, *p* < 0.001). These results were reproducible in the matched sample (Table S2).

### 3.3. Treatments Between Groups

All patients in the present study were treated with CS following the recommendations of the RECOVERY trial [3]. TCZ was usually prescribed with one single dose of TCZ, but additional doses were prescribed in a few patients. There was no difference in the use of TCZ between the groups. According to our hospital protocol, RDSV was prescribed if the patient attended within the first 5 days after the onset of symptoms; therefore, it is logical to find differences in its use between the groups (50.2% vs. 20.8%, *p* < 0.001) (Table S3).

Most patients received LMWH, although there were differences between the groups with respect to prophylactic, intermediate, or full doses.

These results were reproducible in the matched sample (Table S3).

### 3.4. Outcomes Between Groups

Overall, it does appear that in-hospital mortality in patients who received CS in the first week of symptoms was higher (29% vs. 12.8%, *p* < 0.001). They also had a higher requirement for NIMV (30.7% vs. 20.8%, *p* = 0.006), IMV (19.1% vs. 12.8%, *p* = 0.039), and ICU admission (27% vs. 18.4%, *p* = 0.014) (Table 2). These in-hospital mortality outcomes were similar regardless of the number of high-risk criteria (Tables S4 and S5) and were reproducible in the matched sample (Table 2).

### 3.5. Risk Factors for In-Hospital Mortality

In the multivariate study in the matched sample, the following risk factors were associated with higher in-hospital mortality: age (OR = 1.06, *p* < 0.001); Charlson index (OR = 1.34, *p* = 0.001); tachypnea > 20 bpm (OR = 2.58, *p* < 0.001); >3 high-risk criteria of inflammation (OR = 1.94, *p* = 0.012); and CS onset in the first week of symptoms (OR = 2.17, *p* = 0.004). We found, as protective factors, a higher PAFI (OR = 0.99, *p* < 0.001) and the use of RDSV (OR = 0.53, *p* = 0.021) (Table 3).

However, in patients with 1–2 high-risk criteria, the onset of CS in the first week did not reach statistical significance (OR = 2.41, *p* = 0.067). Regardless, a trend towards higher mortality was evident. In patients with three or more high-risk criteria of inflammation, the onset of CS in the first week of symptoms clearly disappeared as a risk factor for mortality (OR = 0.89, *p* = 0.799) (Table 4).

## 4. Discussion

Our study shows that the use of CS in the first week of symptoms is not a risk factor for mortality. Although the raw data appear to suggest that there is higher mortality when starting corticosteroids in the first week, when this variable was introduced in a multivariate model stratified by analytical inflammation, statistical significance was not reached. We can speak of a trend in the case of the group of patients with 1–2 inflammation criteria. Perhaps in this subgroup, and with a larger sample, statistical significance could be reached. In the subgroup of patients with three or more inflammation criteria, it is clearly not a risk factor for mortality.

Our results in the lower inflammation group align with studies indicating that corticosteroids may be detrimental in the viral phase [16,17]. Bahl et al. found no clear benefit in initiating corticosteroids within the first 72 h of admission. In the RECOVERY trial, the receipt of dexamethasone was associated with a reduction in 28-day mortality among those who had experienced symptoms for more than 7 days but not among those with a more recent symptom onset [3]. The 16% increase in mortality in the first-week group with 1–2 inflammation criteria compared to the second-week group reinforces the detrimental character of corticosteroids in this group. Despite the lack of data on viral clearance in our study, controlled studies have shown that corticosteroids do not slow viral clearance [19]. Therefore, the explanation for these results does not seem to be so much linked to viral clearance as to the degree of inflammation existing at the time of initiating corticosteroids. In all these studies, a timeline is mentioned but there are few detailed data on the analytical inflammation of the patients included.

In addition, despite being admitted during the first week of symptoms and therefore during the viral phase, our patients of the first-week group had a high level of systemic inflammation and respiratory failure, equal or higher than those of the second-week group. Therefore, we observed a group with a more rapid and aggressive course of SARS-CoV-2, an aspect that has already been evidenced in clinical practice. Notable differences in several comorbidities were observed between the groups, with several being more prevalent in the first-week group. We cannot rule out the possibility that greater comorbidity translated into greater fragility and a greater sense of severity in these patients and that this was the reason for the earlier administration of the CS. Whether the response to CS may have been influenced by the different comorbidities is a question that is on the table but cannot be answered by the present study.

The multivariate study shows that the use of CS is not an independent risk factor for mortality in this sample of highly inflamed patients. This fact is independent of the time since onset. This is in line with the results demonstrated in previous studies in patients with moderate to severe COVID-19, in which the mortality rate decreased. In the RECOVERY study, it was the group with the greatest impact on mortality [3]. In a recent meta-analysis, it was this group that had the lowest 28-day mortality (RR at 0.85; 95% CI: 0.76–0.95; *p* = 0.004) [24]. In another meta-analysis of critically ill patients, corticosteroids demonstrated reduced mortality regardless of the time of initiation of the corticosteroids [22]. This supports the therapeutic decision not to delay the initiation of corticosteroids in patients with a high degree of inflammation and respiratory involvement, regardless of whether we think that they are in the viral phase of symptom onset. According to the RECOVERY clinical trial, one would expect that, in patients with a higher degree of inflammation, CS would have reached statistical significance as a protective factor for mortality. In our study, we found an OR = 0.89, which was far from statistical significance. It should be remembered that the RECOVERY study [3] does not provide details of the analytical inflammation described in our study; therefore, they are not entirely comparable. It should also be borne in mind that, in very inflamed patients, CS alone, without other immunosuppressants, such as TCZ, may not be sufficient [30].

The study has some obvious strengths. It is a very homogeneous sample of patients treated with the standard dexamethasone regimen published in the RECOVERY study [3]. In addition, the analytical data for PAFI and the five inflammatory parameters are well-detailed. Conducting studies on COVID-19 without knowing the degree of inflammation of the patients leads to erroneous conclusions and not fully understanding what we are talking about. This is the great strength of our study. Our study was performed at a single tertiary center with a mostly Caucasian population. Regardless of the type of hospital, we believe that our results are generalizable to any patient (preferably Caucasian) with COVID-19 with high-risk analytic inflammation according to the previous definition of analytic inflammation.

There are also some limitations. The first is the retrospective observational nature of the study. Second, the disease onset data are based on the temporal perception of the patients. In this sense, there may be some variability; however, in any case, this would affect both groups. Third, some differences between the groups might have influenced the results. For instance, the group under CS in the first week was older, with a higher degree of dependency, and a higher Charlson index. A PSM was performed to minimize these differences; however, matching was not achieved (likely due to the small sample). This could have influenced the results of an absolute higher ratio of mortality in this group. Unlike these inconveniences, the early onset of CS was not found to be a predictor of higher mortality in the subgroup of patients with a very high-risk category of inflammation. This reinforces the idea that the degree of inflammation prevails at the time of making the decision regarding the onset of the immunosuppressive treatment rather than the time from onset. Fourth, given that vaccination in Spain began in January 2021, there may have been some patients included in the study who had received one dose of vaccination against SARS-CoV-2. We do not have the vaccination data in our dataset; this could have slightly affected the results. In any case, we believe that there were few, if any, vaccinated patients included in the study.

Why some patients become inflamed faster and the correlation of viral load with the degree of inflammation or response to immunosuppressive treatments are interesting questions for future research.

## 5. Conclusions

This study investigated the impact of early corticosteroid (CS) administration (within the first week) in COVID-19 patients. While the initial findings suggested increased mortality with early CS use, further analysis considering the degree of inflammation did not demonstrate a significant detrimental effect. These results highlight the need for a nuanced approach to CS therapy in the treatment of COVID-19 that carefully weighs the risks and benefits based on individual patient characteristics and the severity of the inflammation.

## Figures and Tables

**Table 1:** General data between groups.

	Whole Cohort			Matched Sample		
	1st Week	2nd Week	*p*-Value	1st Week	2nd Week	*p*-Value
n	293	288		288	288	
Age, median [IQR]	70.8 [59.2–79.5]	62.7 [53.2–73.1]	<0.001	71.7 [59.6–79.6]	62.7 [53.2–73.1]	<0.001
Gender (males), n (%)	195 (66.6)	193 (67)	0.906	190 (66)	193 (67)	0.791
Race, n (%)			0.002			0.002
Caucasian	244 (83.3)	218 (75.7)		242 (84)	218 (75.7)	
Hispanic	29 (9.9)	57 (19.8)		28 (9.7)	57 (19.8)	
Black	5 (1.7)	0		5 (1.7)	0	
Others	15 (5.1)	13 (4.5)		13 (4.5)	13 (4.5)	
Days from onset to admission, median [IQR]	5 [4–7]	10 [9–11]	<0.001	5 [4–7]	10 [9–11]	<0.001
BMI, median [IQR]	29.3 [26.9–33.5]	30.1 [26.9–33.7]	0.405	29.3 [26.9–33.4]	30.1 [26.9–33.7]	0.374
Degree of dependency, n (%)			0.003			0.002
Moderate-to severe	35 (11.9)	12 (4.2)		35 (12.2)	12 (4.2)	
Arterial hypertension, n (%)	189 (64.5)	143 (49.7)	<0.001	188 (65.3)	143 (49.7)	<0.001
Dyslipidemia, n (%)	148 (50.5)	114 (39.6)	0.008	146 (50.7)	114 (39.6)	0.007
Diabetes mellitus, n (%)	82 (28)	59 (20.5)	0.035	82 (28.5)	59 (20.5)	0.026
Ischaemic cardiopathy, n (%)	19 (6.5)	20 (6.9)	0.825	19 (6.6)	20 (6.9)	0.868
Dementia, n (%)	14 (4.8)	12 (4.2)	0.722	14 (4.9)	12 (4.2)	0.688
Chronic heart failure, n (%)e	23 (7.8)	8 (2.8)	0.007	23 (8)	8 (2.8)	0.006
Chronic liver disease, n (%)	10 (3.4)	7 (2.4)	0.482	10 (3.5)	7 (2.4)	0.460
Severe chronic renal failure, n (%)	12 (4.1)	4 (1.4)	0.046	12 (4.2)	4 (1.4)	0.043
Cancer, n (%)	20 (6.8)	12 (4.2)	0.160	20 (6.9)	12 (4.2)	0.146
COPD, n (%)	28 (9.6)	16 (5.6)	0.068	28 (9.7)	16 (5.6)	0.060
Charlson index, median [IQR]	1 [0–2]	0 [0, 1]	<0.001	1 [0–2]	0 [0, 1]	<0.001

BMI: body mass index. IQR: interquartile range. COPD: chronic obstructive pulmonary disease. Severe chronic renal failure: creatinine > 300 mg/dL or dialysis. Dyslipidemia included elevated LDL and/or hypertriglyceridemia.

**Table 2:** Outcomes between groups.

	Whole Cohort			Matched Sample		
	1st Week	2nd Week	*p*-Value	1st Week	2nd Week	*p*-Value
Primary outcome n (%)						
In-hospital mortality	85/293 (29)	37/288 (12.8)	<0.001	85/288 (29.5)	37/288 (12.8)	<0.001
1–2 criteria	50/209 (23.9)	18/214 (8.4)	<0.001	50/206 (24.3)	18/214 (8.4)	<0.001
≥3 criteria	35/84 (41.7)	19/74 (25.7)	0.034	35/82 (42.7)	19/74 (25.7)	0.026
Secondary outcomes n (%)						
HFNC	128 (43.7)	107 (37.2)	0.109	128 (44.4)	107 (37.2)	0.075
NIMV	90 (30.7)	60 (20.8)	0.006	90 (31.3)	60 (20.8)	0.004
IMV	56 (19.1)	37 (12.8)	0.039	56 (19.4)	37 (12.8)	0.031
ICU admission	79 (27)	53 (18.4)	0.014	79 (27.4)	53 (18.4)	0.010

HFNC: high-flow nasal cannula. NIMV: non-invasive mechanical ventilation. IMV: invasive mechanical ventilation. ICU: intensive care unit.

**Table 3:** Risk factors of in-hospital mortality in the matched sample.

	Univariate Analysis		Multivariate Analysis	
	OR (95%CI)	*p*-Value	OR (95%CI)	*p*-Value
Age/year	1.05 (1.04–1.07)	<0.001	1.06 (1.04–1.08)	<0.001
Gender (female)	0.83 (0.54–1.28)	0.402	0.85 (0.51–1.42)	0.540
Days from onset to admission	0.88 (0.83–0.94)	<0.001	1.00 (0.89–1.12)	0.995
BMI	0.96 (0.93–0.99)	0.046	0.98 (0.93–1.02)	0.262
Charlson index	1.47 (1.29–1.69)	<0.001	1.34 (1.13–1.57)	0.001
PaO_2_/FiO_2_	0.99 (0.99–0.99)	<0.001	0.99 (0.99–0.99)	<0.001
Respiratory rate > 20 bpm	2.62 (1.66–4.13)	<0.001	2.58 (1.53–4.36)	<0.001
Degrees of inflammation				
≥3 high-risk criteria	2.74 (1.80–4.17)	<0.001	1.94 (1.15–3.25)	0.012
Initiation of corticosteroids from onset				
1st week	2.84 (1.85–4.36)	<0.001	2.17 (1.28–3.69)	0.004
Remdesivir	0.63 (0.41–0.97)	0.037	0.53 (0.31–0.91)	0.021
Tocilizumab	1.02 (0.64–1.61)	0.946		

BMI: body mass index.

**Table 4:** Risk factors of in-hospital mortality in the matched sample according to the number of high-risk criteria.

Patients with 1–2 High-Risk Criteria				
	Univariate Analysis		Multivariate Analysis	
	OR (95%CI)	*p*-Value	OR (95%CI)	*p*-Value
Age/year	1.05 (1.03–1.07)	<0.001	1.05 (1.03–1.08)	<0.001
Gender (female)	0.62 (0.35–1.10)	0.105		NS
Days from onset to admission	0.86 (0.79–0.94)	0.001		NS
BMI	1.01 (0.96–1.06)	0.751		
Charlson index	1.42 (1.20–1.68)	<0.001	1.26 (1.03–1.55)	0.028
PaO_2_/FiO_2_	0.99 (0.99–0.99)	<0.001	0.99 (0.99–0.99)	0.001
Respiratory rate > 20 bpm	3.56 (1.91–6.65)	<0.001	4.28 (2.13–8.61)	<0.001
Initiation of corticosteroids from onset				
1st week	3.49 (1.96–6.22)	<0.001	2.41 (0.94–6.18)	0.067
Remdesivir	0.77 (0.45–1.34)	0.358		
Tocilizumab	1.22 (0.68–2.22)	0.505		
**Patients with ≥3 high-risk criteria**				
	**Univariate analysis**		**Multivariate analysis**	
	**OR (95%CI)**	***p*-value**	**OR (95%CI)**	***p*-value**
Age/year	1.07 (1.04–1.11)	<0.001	1.08 (1.04–1.12)	<0.001
Gender (male)	1.93 (0.92–4.05)	0.083		NS
Days from onset to admission	0.92 (0.83–1.02)	0.104		
BMI	0.88 (0.81–0.95)	0.002	0.86 (0.78–0.95)	0.003
Charlson index	1.58 (1.23–2.02)	<0.001	1.50 (1.11–2.01)	0.007
PaO_2_/FiO_2_	0.99 (0.99–0.99)	0.005	0.99 (0.98–0.99)	<0.001
Respiratory rate > 20 bpm	1.36 (0.66–2.80)	0.406		
Initiation of corticosteroids from onset				
1st week	2.16 (1.09–4.26)	0.027	0.89 (0.37–2.14)	0.799
Remdesivir	0.54 (0.25–1.17)	0.117		
Tocilizumab	0.63 (0.30–1.34)	0.232		

BMI: body mass index. NS: not significant.

## Data Availability

Datasets generated during the current study are available from the corresponding author on reasonable request.

## Supplementary Materials

The following supporting information can be downloaded at: https://www.mdpi.com/article/10.3390/medicina61020233/s1, Table S1: Symptoms and physical examination upon admission between groups. Table S2: Treatments between groups. Table S3: Outcomes between groups in patients with 1–2 high-risk criteria. Table S4: Outcomes between groups in patients with 3–4–5 high-risk criteria. Table S5. Outcomes between groups in patients with 3-4-5 high-risk criteria.
