# Supplementary material for: Should the Start of Immunosuppressive Treatment for COVID-19 Rely upon the Degree of Inflammation or the Time from Onset?
Source: Medicina (Kaunas). 2025 Jan 27;61(2):233. doi: 10.3390/medicina61020233 (PMC11857332; doi:10.3390/medicina61020233)
Supplement: Supplementary file 1 [file medicina-61-00233-s001.zip › medicina-3409529-supplementary.pdf]

Supplementary Materials

Table S1. Symptoms and physical examination upon admission between groups

	Whole cohort			Matched sample		
	**1ST week**	**2nd week**	**p-value**	**1ST week**	**2nd week**	**p-value**
Cough, n (%)	192 (65.5)	223 (77.4)	**0.006**	188 (65.2)	223 (77.4)	**0.005**
Arthromyalgias, n (%)	56 (19.1)	92 (31.9)	**<0.001**	54 (18.8)	92 (31.9)	**<0.001**
Ageusia, n (%)	39 (13.3)	64 (22.2)	**0.005**	39 (13.5)	64 (22.2)	**0.007**
Anosmia, n (%)	32 (10.9)	55 (19.1)	**0.006**	31 (10.8)	55 (19.1)	**0.005**
Sore throat, n (%)	19 (6.5)	22 (7.6)	0.587	19 (6.6)	22 (7.6)	0.627
Headache, n (%)	36 (12.3)	56 (19.4)	**0.018**	36 (12.5)	56 (19.4)	**0.023**
Fever, n (%)	225 (76.8)	243 (84.4)	**0.033**	221 (76.8)	243 (84.4)	**0.028**
Dyspnea, n (%)	200 (68.3)	187 (64.9)	0.395	198 (68.8)	187 (64.9)	0.330
Diarrhea, n (%)	77 (26.3)	105 (36.5)	**0.008**	76 (26.4)	105 (36.5)	**0.009**
Vomiting, n (%)	15 (5.1)	17 (5.9)	0.679	15 (5.2)	17 (5.9)	0.716
Abdominal pain, n (%)	11 (3.8)	11 (3.8)	0.967	11 (3.8)	11 (3.8)	1.000
Heart rate, bpm median [IQR]	89 [79-100]	95 [82-104]	**0.001**	89 [79-100]	95 [82-104]	**0.001**
Respiratory rate >20 bpm, n (%)	175 (59.7)	170 (59)	0.896	173 (60.1)	170 (59)	0.799

IQR: interquartile range.

Table S2. Lab tests upon admission between groups

	Whole cohort			Matched sample		
	**1ST week**	**2nd week**	**p-value**	**1ST week**	**2nd week**	**p-value**
PaO2/FiO2, median [IQR]	290 [213-338]	311 [267-357]	**<0.001**	290 [211-333]	311 [267-357]	**<0.001**
Lymphocytes $x10^6$/L, median [IQR]	840 [580-1,175]	900 [683-1,235]	**0.033**	830 [580-1,178]	900 [683-1,235]	**0.028**
CRP mg/L, median [IQR]	98 [57-169]	112 [60-172]	0.274	97 [57-170]	112 [60-172]	0.257
LDH U/L, median [IQR]	345 [274-441]	348 [283-446]	0.657	345 [274-441]	348 [283-446]	0.673
Ferritin mcg/L, median [IQR]	931 [490-1,588]	910 [439-1,780]	0.742	927 [485-1,551]	910 [439-1,780]	0.602
D-dimer ng/mL, median [IQR]	400 [260-847]	309 [250-523]	**<0.001**	405 [262-855]	309 [250-523]	**<0.001**
1-2 high-risk criteria, n (%)	209 (71.3)	214 (74.3)	0.420	206 (71.5)	214 (74.3)	0.453
≥ 3 high-risk criteria, n (%)	84 (28.7)	74 (25.7)	0.420	82 (28.5)	74 (25.7)	0.453

CRP: C-reactive protein. LDH: lactate dehydrogenase. IQR: interquartile range.

Table S3. Treatments between groups

	Whole cohort			Matched sample		
	**1ST week**	**2nd week**	**p-value**	**1ST week**	**2nd week**	**p-value**
Remdesivir	147 (50.2)	60 (20.8)	**<0.001**	147 (51)	60 (20.8)	**<0.001**
Tocilizumab	75 (25.6)	73 (25.3)	0.945	72 (25)	73 (25.3)	0.924
LMWH			**0.035**			**0.026**
No	28 (9.6)	25 (8.7)		28 (9.7)	25 (8.7)	
Low doses	146 (49.8)	174 (60.4)		142 (49.3)	174 (60.4)	
Intermediate doses	71 (24.2)	61 (21.2)		70 (24.3)	61 (21.2)	
Full doses	48 (16.4)	28 (9.7)		48 (16.7)	28 (9.7)	

LMWH: Low-molecular-weight heparin.

Table S4. Outcomes between groups in patients with 1-2 high-risk criteria

	Whole cohort			Matched sample		
	**1ST week**	**2nd week**	**p-value**	**1ST week**	**2nd week**	**p-value**
Primary outcome n (%)						
In-hospital mortality	50 (23.9)	18 (8.4)	**<0.001**	50 (24.3)	18 (8.4)	**<0.001**
Secondary outcomes n (%)						
HFNC	85 (40.7)	63 (29.4)	**0.015**	85 (41.3)	63 (29.4)	**0.011**
NIMV	50 (23.9)	36 (16.8)	0.070	50 (24.3)	36 (16.8)	0.059
IMV	28 (13.4)	17 (7.9)	0.069	28 (13.6)	17 (7.9)	0.061
ICU admission	40 (19.1)	30 (14)	0.157	40 (19.4)	30 (14)	0.138

HFNC: high-flow nasal cannula. NIMV: non-invasive mechanical ventilation. IMV: invasive mechanical ventilation. ICU: intensive care unit

Table S5. Outcomes between groups in patients with 3-4-5 high-risk criteria

	Whole cohort			Matched sample		
	**1ST week**	**2nd week**	**p-value**	**1ST week**	**2nd week**	**p-value**
Primary outcome n (%)						
In-hospital mortality	35 (41.7)	19 (25.7)	**0.034**	35 (42.7)	19 (25.7)	**0.026**
Secondary outcomes n (%)						
HFNC	43 (51.2)	44 (59.5)	0.297	43 (52.4)	44 (59.5)	0.378
NIMV	40 (47.6)	24 (32.4)	0.052	40 (48.8)	24 (32.4)	**0.038**
IMV	28 (33.3)	20 (27)	0.390	28 (34.1)	20 (27)	0.336
ICU admission	39 (46.4)	23 (31.1)	**0.049**	39 (47.6)	23 (31.1)	**0.036**

HFNC: high-flow nasal cannula. NIMV: non-invasive mechanical ventilation. IMV: invasive mechanical ventilation. ICU: intensive care unit
